# Histone acetyltransferase inhibition reverses opacity in rat galactose-induced cataract

**DOI:** 10.1371/journal.pone.0273868

**Published:** 2022-11-23

**Authors:** Masaya Nagaya, Risa Yamaoka, Fumito Kanada, Tamotsu Sawa, Masaru Takashima, Yoshihiro Takamura, Masaru Inatani, Masaya Oki

**Affiliations:** 1 Department of Industrial Creation Engineering, Graduate School of Engineering, University of Fukui, Fukui, Japan; 2 Department of Ophthalmology, Faculty of Medical Sciences, University of Fukui, Fukui, Japan; 3 Life Science Innovation Center, University of Fukui, Fukui, Japan; Oakland University, UNITED STATES

## Abstract

Cataract, a disease that causes opacity of the lens, is the leading cause of blindness worldwide. Cataracts secondary to diabetes are common, even in young patients, so they are of significant clinical importance. Here, we used an *ex vivo* model of galactose-induced cataracts in the rat lens to investigate the therapeutic effects of histone acetyltransferase (HAT) inhibitors. Among the tested HAT inhibitors, TH1834 was the only one that could reverse most of the opacity once it had formed in the lens. Combination treatment with C646/CPTH2 and CBP30/CPTH2 also had therapeutic effects. In lens cross-sections, vacuoles were present in the tissue of the cortical equatorial region of untreated cataract samples. In treated cataract samples, lens tissue regenerated to fill the vacuoles. To identify the genes regulated by HAT inhibitors, qRT-PCR was performed on treated and untreated cataract samples to determine candidate genes. Expression of *Acta1* and *Stmn4*, both of which are involved in the cytoskeleton, were altered significantly in C646+CPTH2 samples. Expression of *Emd*, a nuclear membrane protein, and *Prtfdc1*, which is involved in cancer cell proliferation, were altered significantly in CBP30+CPTH2 samples. *Acta1*, *Acta2*, *Arrdc3*, *Hebp2*, *Hist2h2ab*, *Pmf1*, *Ppdpf*, *Rbm3*, *RGD1561694*, *Slc16a6*, *Slfn13*, *Tagln*, *Tgfb1i1*, and *Tuba1c* in TH1834 samples were significantly altered. These genes were primarily related to regulation of cell proliferation, the cytoskeleton, and cell differentiation. Expression levels increased with the onset of cataracts and was suppressed in samples treated with HAT inhibitors.

## Introduction

Cataract, characterized by clouding of the lens, vision loss, and eventual blindness, accounts for up to 75% of all cases of blindness worldwide [1]. Currently, there are no therapeutic agents available for cataract; the only treatment available is surgical insertion of an artificial lens. However, due to the unavailability of surgery in developing countries, and the risk of secondary cataracts in young patients over time, treatment with eye drops is clinically desirable [2, 3]. In diabetic persons, cataracts are induced by hyperglycemia, and the relationship between cataracts, diabetes, and galactosemia has been well-studied [4]. The incidence of diabetes is increasing worldwide [5], and elucidating the mechanisms underlying diabetic cataract formation is necessary if we are to develop targeted approaches to treatment.

Three major causes of diabetic cataract have been proposed: increased osmolarity with activation of the polyol pathway [6, 7], aggregation and opacification of lens proteins due to glycation [8], and apoptosis of lens epithelial cells [9]. In rats, galactose-induced cataracts, like diabetic cataracts, are thought to be caused by polyol accumulation, which activates apoptosis [9, 10]. Inhibitors of aldose reductase, which is involved in the polyol pathway, inhibit apoptosis, suggesting that sugar alcohol accumulation and apoptosis could be closely related [9]. In addition, expression of p21, which is involved in inhibition of cell proliferation, also increases as galactose-induced cataracts progress [11]. The balance between cell cycle progression and inhibition is important for maintenance of lens transparency. Activation of apoptosis and decreased lens epithelial cell (LEC) density have also been observed in human diabetic cataracts [10, 12]. Organelles within lens fiber cells in the lens cortex are degraded by mechanisms specific to the lens [13], and maintenance of epithelial cell homeostasis has a profound effect on the whole lens. Many studies have sought to prevent diabetic cataract based on these mechanisms [14–19], but few have aimed to develop therapeutics to alleviate previously formed cataracts. Aldose reductase inhibitors prevent and treat galactose-induced cataracts [20], but are not expected to be clinically applicable due to their instability and toxic side effects [21]. The therapeutic effect of lanosterol has been reported in rabbits and dogs with age-related nuclear cataracts [22]. Aggregation of crystallin proteins, which make up the lens, is one cause of cataract formation [23]. Lanosterol is amphiphilic, and becomes water-soluble by acting on the hydrophobic sites of protein aggregates, which may be important for reducing cataract formation. Stabilizing alpha-crystallin, a molecular chaperone that maintains the solubility of lens proteins, can treat age-related cataracts in mice [24], so resolving protein aggregation is important for cataract treatment. However, there is no proof that lanosterol actually binds to lens proteins and dissolves cataracts [25], and it does not alleviate nuclear cataracts in humans [26]. Therefore, further research is needed before it can be used as a therapeutic agent. Also, these are studies of nuclear cataracts rather than cortical cataracts, which are triggered by diabetes and other conditions [22]. Therefore, elucidating the regulatory mechanisms underlying diabetic cataract formation and then exploiting these mechanisms to develop new therapeutic targets is desirable.

Regulation of gene expression through histone acetylation, an epigenetic regulatory mechanism, has been implicated in a variety of diseases, including diabetes [27–29]. Histone acetylation loosens aggregated inactive chromatin and activates transcription of genes in the surrounding regions [30]. Histone acetyltransferases (HATs) and histone deacetylases (HDACs) are being investigated as targets for drug discovery [31–34]. In a previous study, we found that HAT inhibitors prevent, and HDAC inhibitors accelerate, galactose-induced cataract formation in rats [15]. Furthermore, microarray analysis identified Plk3, which is repressed by HATs and activated by HDACs, as being important for cataract formation. HATs belong to the GNAT (Gcn5-related N-acetyltransferase) family, the MYST (Moz, Ybf2/Sas3, Sas2, Tip60) family, the CBP (CREB-binding protein)/p300 family, the TAF1 (TATA-Box Binding Protein Associated Factor 1) family, and others [35]. The GNAT family consists of proteins such as Gcn5 and p/CAF, and the MYST family consists of proteins such as Tip60 and MOZ, which are classified according to function, structure, and target [35]. Specific inhibitors of these proteins, including CPTH2, an inhibitor of Gcn5 [36]; Embelin, a p/CAF inhibitor [37]; TH1834, a Tip60 inhibitor [37]; and C646 and CBP30, both CBP/p300 inhibitors [37], prevent galactose-induced cataracts [15]. This indicates that histone acetylation plays an important role in development and progression of galactose-induced cataracts. Several studies have investigated the relationship between histone acetylation and cataracts, including one report showing that culturing rabbit lenses with anacardic acid, a HAT inhibitor, causes surface clouding. Culturing lenses in medium containing both anacardic acid and trichostatin A, an HDAC inhibitor, inhibited formation of lens opacity [38]. Furthermore, TGF-β-induced lens clouding can be prevented by tubacin, which inhibits HDAC6 [39]. In these models, the cataract occurs primarily in the posterior capsule of the lens. The mechanism is clearly different from that of the galactose-cataract model, in which vacuoles occur in the cortical area, and there are no reports to date of HDAC inhibitors preventing diabetic cataracts.

In the present study, we screened HAT inhibitors using a rat galactose-induced *ex vivo* cataract experimental system to determine whether they can alleviate cataracts once formed. A specific HAT inhibitor was an effective treatment for galactose-induced cataracts. We then examined HAT-regulated genes by qRT-PCR, and identified genes that were altered in opaque lenses. These results indicate that HAT inhibitors, and inhibition of their downstream target genes, may be effective therapeutic agents that reduce lens opacity in diabetic cataracts.

## Materials and methods

### *Ex vivo* assays

Six-week-old male Sprague Dawley (SD) rats were purchased from Sankyo Lab Services. Rats were euthanized by CO_2_ asphyxiation. Both lenses were removed from each animal, placed in 12-well Petri dishes (37°C, 5% CO_2_), and cultured in 2 ml M199 medium containing 0.1% BSA and 30 mM galactose [15]. After incubation until a white opacity formed, the medium covering both lenses was replaced with fresh medium, and one lens from each pair was treated with HAT inhibitors dissolved in DMSO (C646, Sigma, final concentration 40 μM), CBP30 (Cayman Chemical, final concentration 10 μM), CPTH2 (Cayman Chemical, final concentration 80 μM), and TH1834 (Axon MedChem, final concentration 50 μM). Inhibitors were used alone or as a mixture. The other lens from each pair was exposed to DMSO alone (vehicle control). Control samples were incubated with sterile water instead of galactose. The experiments were approved by Animal Research Committee, University of Fukui (Application number: 28091), and all experimental procedures involving animal were conducted in accordance with the University of Fukui guidelines.

### Microscopic observations

Photographs of the lenses were taken using an SZX12 stereomicroscope in combination with a DP58 camera (Olympus), as previously reported [15]. Images were taken with the lens immersed in 7 mL of PBS in a 35 mm dish. Opacity in the cortical area of the lens (the area 20% from the periphery looking at the lens from above) was measured in terms of brightness (0–255) using ImageJ, and a weighted average was calculated.

### RNA extraction, cDNA preparation, and real-time qRT-PCR

RNA extraction and real-time qRT-PCR were performed as previously reported [15]. Target genes and Gapdh were amplified using SYBR Green master mix (Applied Biosystems) and gene specific primers for quantitative analysis of mRNA expression by real-time PCR. The primers used are listed in the Supplementary S1 Table. Expression levels were normalized to those of *Gapdh*. Statistical analysis of RT-qPCR results was performed using Microsoft Excel Office, and a two-tailed unpaired Student’s t-test was used to evaluate differences between the galactose group and the control or drug-treated group. *P* < 0.05 was considered statistically significant.

### Tissue sectioning and staining

After incubation, lenses were immersed in formalin-glutaraldehyde (FG) solution (a mixture of 10% formalin and 25% glutaraldehyde at a ratio of 1:9) for 3 days at 4°C, as described previously [15]. The FG solution was changed every other day. The lenses were then immersed in 10% formalin solution at room temperature for at least 1 day. Samples were then transported to New Histo. Science Laboratory Co., Ltd. for preparation of paraffin sections. Stained sections were examined at 100x using an IX70 microscope (OLYMPUS).

### Microarray data analysis

Microarray analysis was performed on 7 samples: control lenses cultured in medium without galactose (n = 1), lenses cultured for 4 days in medium containing galactose (n = 3), and lenses cultured for 6 days in medium containing galactose (n = 3). A GeneChip Rat Gene 2.0 ST array (Thermo Fisher Scientific) was used for these experiments, as described previously [15]. Preprocessing and analysis were performed using R software. Unnamed genes, and genes with signal values <5, in all samples were eliminated. Heat maps and principal component analysis maps were generated for genes whose expression was predominantly increased (*P* < 0.1) in any of the six galactose-only samples, and decreased (*P* < 0.1) in any of the three treatment samples, relative to the control. The data are available under accession number GSE186248 at https://www.ncbi.nlm.nih.gov/geo/query/acc.cgi?acc=GSE186248.

## Results

### Effect of HAT inhibitors on the opacity of galactose-induced cataracts in rats

**Fig 1:**
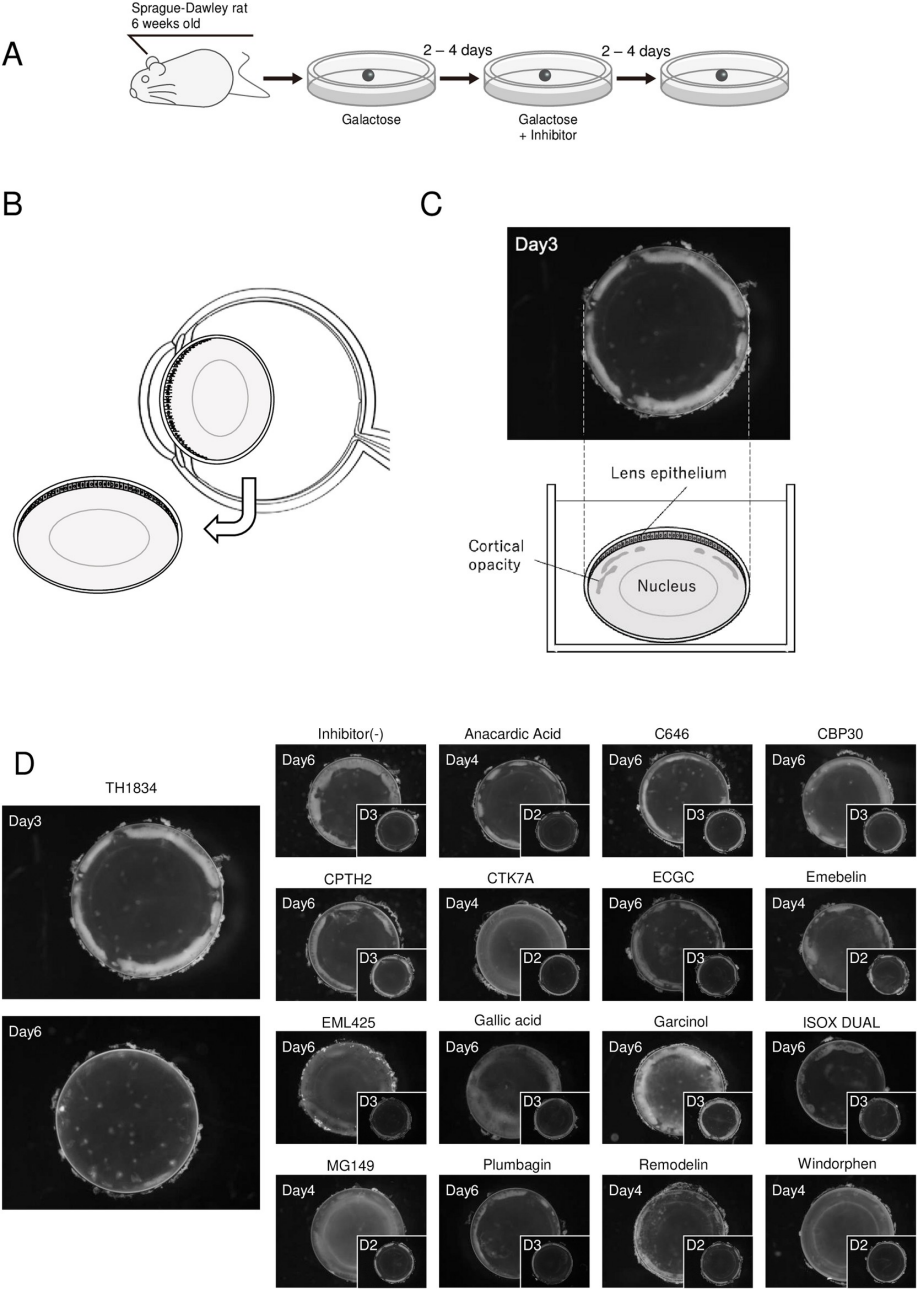
Effect of HAT inhibitors on once-formed galactose cataracts. (A) Schematic illustration of the *ex vivo* experiment. First, cataracts were induced by incubating lenses for 2–4 days in medium containing 30 mM galactose. Next, inhibitors were added to one lens, and DMSO (vehicle control) was added to the contralateral lens. Both lenses were then incubated for another 2–4 days. (B) Position of the eyeball and lens. (C) The lens is observed from above using a stereomicroscope. (D) Microscopic images of lenses incubated with HAT inhibitors. The small photograph on the lower right shows the lens before addition of HAT inhibitor. The number of incubation days is shown in the upper left corner of the photograph. The 16 HAT inhibitors previously identified to have a cataract-preventive effect were used at the same concentrations used previously (S2 Table).

In a previous study, we found that 16 HAT inhibitors prevent diabetes-like cataracts [15]. In the present study, we further examined the therapeutic effects of these 16 HAT inhibitors. First, we removed the crystalline lens from SD rats, cultured them in galactose-containing medium until they developed cortical cataracts of similar opacity, and then added one of the 16 HAT inhibitors shown to prevent cataract formation in previous studies to determine whether these inhibitors could reverse lens opacity once formed (Fig 1A–1D). The concentrations of the inhibitors used in the analysis are shown in S2 Table. A schematic diagram representing the part of the eye shown in the photograph in Fig 1D is shown in Fig 1B and 1C. As a result, TH1834, an inhibitor of Tip60, reduced the opacity of the equator to a large fraction compared with the lens before TH1834 treatment (which had been cultured in galactose-containing medium for 3 days) and the control lens (which had been cultured in galactose-containing medium for 6 days) (Fig 1D). The Day 6 photograph revealed pointed or banded cortical opacity in the periphery of lenses exposed to galactose, where the effect of TH1834 treatment was observed. Pointed and banded cortical opacities are early morphological features of galactose-induced cataracts [40]. The results also suggested that addition of TH1834 eliminated the white opacity once formed. In addition, the lenses cultured with CPTH2, an inhibitor of Gcn5, showed a slight decrease in opacity, although there were individual differences (S1 Fig). Lenses treated with the other 14 inhibitors showed either the presence of cortical equatorial opacity or progressive clouding of the whole lens.

**Fig 2:**
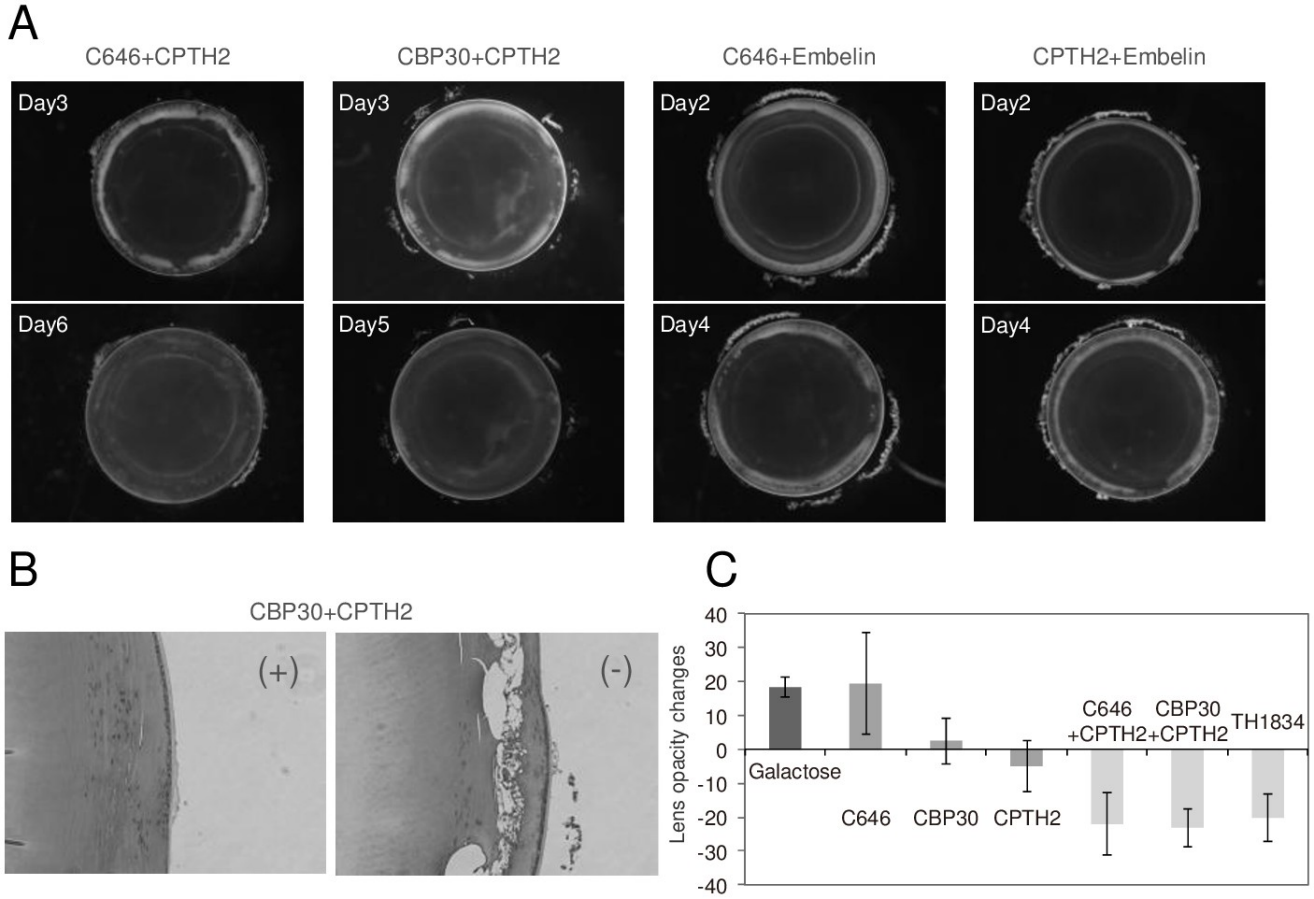
Combined effects of two HAT inhibitors on galactose-induced cataracts. (A) The upper figure shows micrographs taken after incubation without inhibitors, and the lower figure shows micrographs taken after incubation with inhibitors. The number of incubation days is shown in the upper left corner of the photograph. (B) The image on the left (+) shows sections prepared after incubation in galactose-containing medium, followed by addition of CBP30 (10 μM) + CPTH2 (80 μM), and H&E staining. The image on the right (-) shows a section prepared after incubation in galactose-containing medium without inhibitors, followed by H&E staining. (C) Quantification of lens opacity. The opacity of the cortical region of the lens was calculated as previously indicated by subtracting the opacity value measured before addition of the inhibitor from the opacity after addition of the inhibitor [15]. Data are expressed as the mean ± SE. Samples used for quantification are shown in S2 Fig.

Interestingly, our previous study confirmed that inhibitors targeting different HATs prevent cataracts [15]. For example, TH1834 inhibits Tip60, Embelin inhibits p/CAF, CPTH2 inhibits Gcn5, and C646 inhibits p300. Therefore, to investigate the relationship between these HAT inhibitors, we examined whether combination treatment with HAT inhibitors that inhibit different targets could reverse galactose-induced lens opacification. Combination treatment with C646 and CPTH2 almost completely eliminated white opacity of the cortex (Fig 2A and S1 Fig). The white opacity of the cortex was also reduced by CBP30+CPTH2 combination. C646 and CBP30 inhibit the same target, p300. Combination treatment with C646+Embelin or CPTH2+Embelin had no effect.

Next, to observe changes inside the lens, we examined cross-sections of lenses treated with effective HAT inhibitors. Disintegration of the lens cortex was observed upon culture in galactose-containing medium. Disintegration was almost completely eliminated in crystalline lenses co-treated with HAT inhibitors (Fig 2B). In detail, the vacuoles themselves were formed, but appeared to be collapsed and vertical in shape. The space created by cell damage and tissue disorganization in galactose-induced cataracts was normalized by the HAT inhibitor.

To quantitatively evaluate lens opacity, we measured relative changes in opacity by calculating the difference in opacity (the mean luminance value of the equatorial part of the lens cortex) before and after vehicle or inhibitor treatment. Photographs of the samples used for quantification are shown in S2 Fig. Samples treated with vehicle or C646 exhibited comparable increases in opacity (Fig 2C). The opacity of CBP30-treated lenses increased slightly, although not as much as that of vehicle-treated lenses (Fig 2C). The opacity of CPTH2-treated lenses showed a decreasing trend, and the opacities of lenses treated with C646+CPTH2, CBP30+CPTH2, and TH1834 decreased markedly (Fig 2C). Although CPTH2 alone had a weak therapeutic effect on cataracts, co-treatment with either C646 or CBP30, both p300 inhibitors, strengthened the effect. Monotreatment with TH1834 was as effective as the co-treatment.

### Changes in gene expression levels upon cataract formation

**Fig 3:**
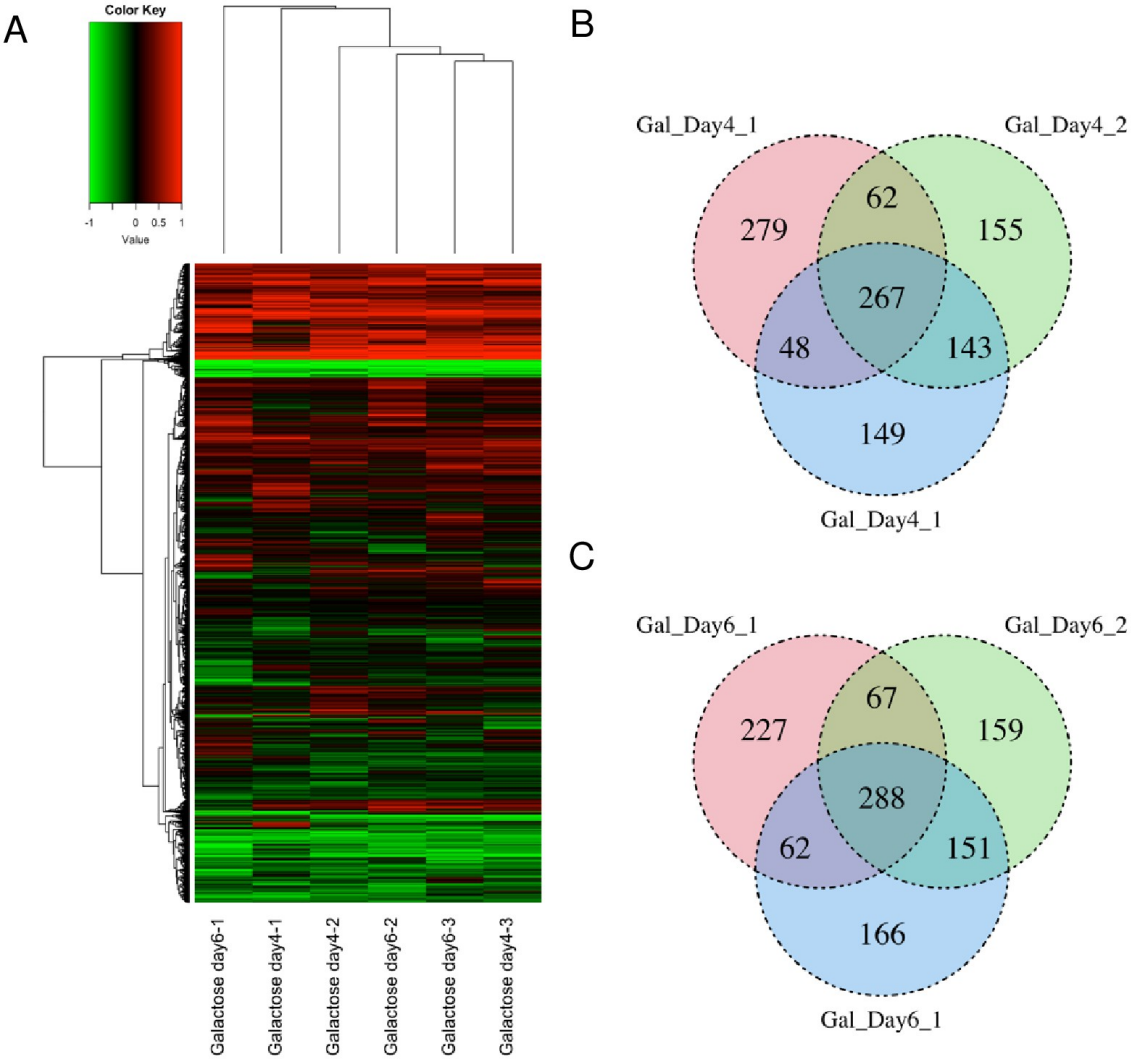
Genes whose expression differs significantly from that of control galactose Day 4 and 6 samples. (A) Heatmap showing gene expression changes between six galactose samples and the control sample. We show the value obtained by subtracting the signal value of the control sample from the signal value of each galactose sample, representing 21,282 differentially expressed genes. (B) Venn diagram showing the relationship among the three groups of genes showing differential expression (*P* < 0.1) between the control sample and each galactose Day 4 sample. (C) Venn diagram showing the relationship among the three groups of genes showing differential expression (*P* < 0.1) between the control sample and each galactose Day 6 sample.

We conducted microarray analysis to identify genes that show altered expression during cataract formation in control samples cultured without HAT inhibitors or galactose, and in lenses cultured in galactose (S2 Fig). There were no significant differences in gene expression profiles between samples cultured for 4 days versus 6 days, although the cloudiness of galactose Day 6 lenses was greater than that of galactose Day 4 lenses (Fig 3A and S2 Fig). Therefore, we analyzed the microarray data in more detail. First, we deleted 13,580 out of 21,282 genes with signal values <5 on Day 4 and Day 6, leaving 7,702 genes. We then examined the relationship between genes showing significantly different expression (*P* < 0.1) between control and galactose-treated lenses (Day 4), and found that 656, 627, and 607 genes were increased or decreased in the three replicates. Using a Venn diagram, we identified 267 genes that were altered in all three galactose Day4 (Fig 3B and S1 Dataset). About 25% of the genes in each replicate (279, 155, and 149 genes) significantly differed from the control in only one replicate. This could be due to inherent experimental error in the microarray analyses, or differences in cataract severity between replicates of the same days of incubation. We analyzed the galactose Day 6 samples in the same way as the Day 4 samples. We found significant differences (*P* < 0.1) in expression of genes between control and galactose-treated lenses (Day 6), identifying 644, 665, and 667 genes in the three replicates. From the Venn diagram, we found that 288 genes were significantly altered in all galactose Day 6 samples (Fig 3C and S1 Dataset). Similar to the results of galactose Day 4, about 25% of the genes in each replicate (227, 159, and 166 genes) were significantly different in only one sample.

Next, to detect genes with relatively small individual changes over time, we extracted genes with a mean signal value variation of >1.5-fold and a p-value <0.05 in control versus galactose Day 4, or galactose Day4 versus galactose Day 6 samples (S3 Table). Most genes (933) were upregulated between control and galactose Day 4, but not between galactose Day 4 and galactose Day 6. Also, expression was upregulated maximally during initial white opacity (Day 4). One gene (*Hspa1b*) changed over time in accordance with cataract progression; specifically, its expression increased (-fold change >1.5) in galactose Day 4 relative to control, and increased further (*P* < 0.05, -fold change >1.5) in galactose Day 6 relative to galactose Day 4. Nine genes (*Tcp11l2*, *Rdh11*, *Rcbtb2*, *Nrip2*, *LOC100911253*, *LOC100362572*, *Hist1h2bh*, *Gtpbp2*, and *Bace1*) were upregulated in galactose Day 4 relative to control, but downregulated from Day 4 to Day 6 (>1.5-fold decrease, *P* < 0.05). Nine genes (*Tprn*, *Tgfb3*, *Sgpp1*, *Rtn4*, *Mir22 Ier3*, *Hamp*, *Egr1*, and *Cd44*) were expressed after the mid-cataract stage, with no increase in expression in control versus galactose Day 4, and increased expression from Day 4 to Day 6 (>1.5-fold increase, *P* < 0.05). These genes could serve as markers for progression and severity of diabetic cataracts.

### Identification of downstream factors regulated by HAT inhibitors

To determine the criteria for extraction of genes affected by HAT inhibitors, we first extracted genes that were altered in galactose-treated lenses and reversed by HAT inhibitors at multiple thresholds (*P* < 0.1, *P* < 0.05, and *P* < 0.01); we then compared the number of genes. We extracted 421 genes with *P* < 0.1, 248 genes with *P* < 0.05, and 109 genes with *P* < 0.01 that were upregulated by galactose and downregulated by HAT inhibitors (S4 Table). We also extracted 412 genes with *P* < 0.1, 221 genes with *P* < 0.05, and 77 genes with *P* < 0.01 that were downregulated by galactose and restored by HAT inhibitor treatment (S4 Table). When we restricted the results to *P* < 0.1, even TH1834 (which showed only a small variation in expression compared with galactose treatment) showed more than a 1.5-fold variation in expression level, suggesting that it may be possible to extract genes affected by HAT inhibitors using these criteria. Therefore, in this analysis, 833 candidate genes with *P* < 0.1 were extracted (S2 and S3 Datasets).

**Fig 4:**
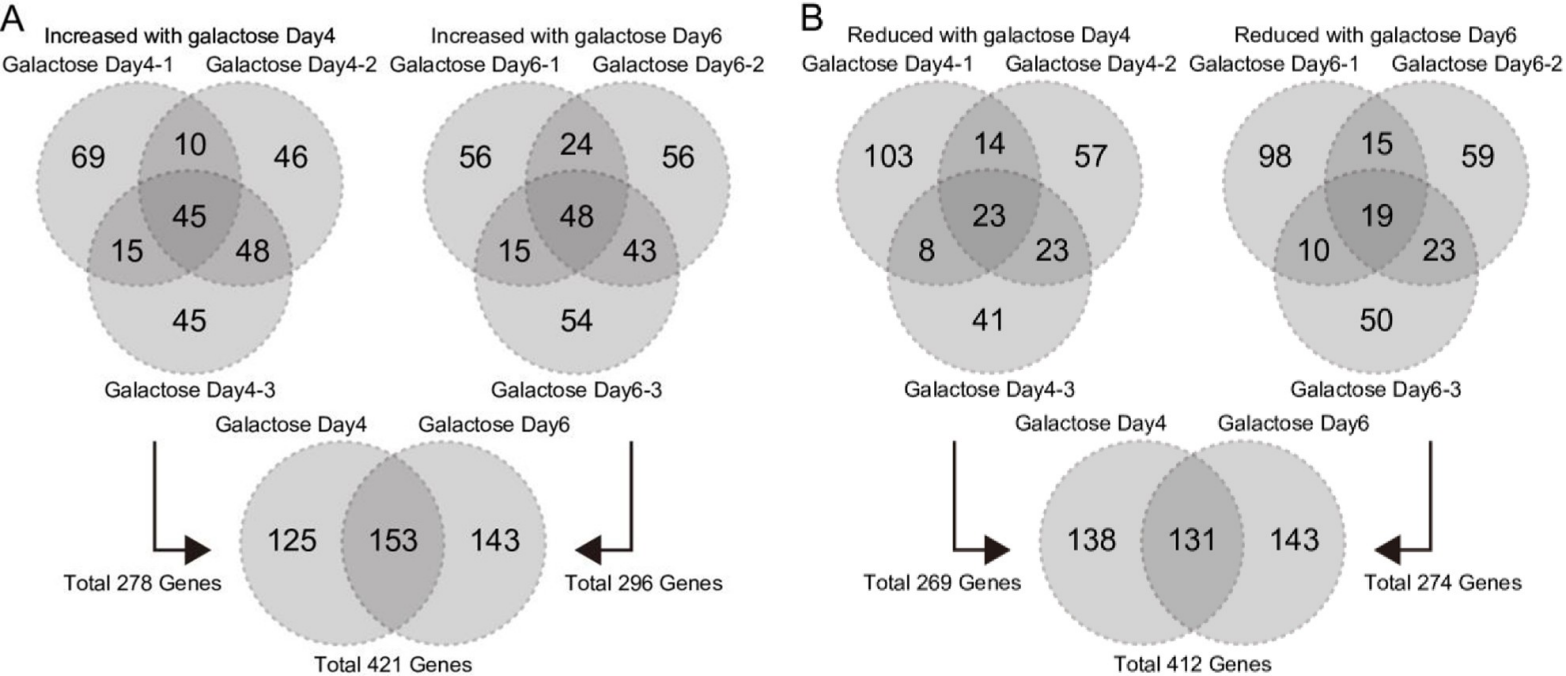
Microarray analysis identified genes whose expression is altered by galactose. (A) Venn diagram of genes upregulated in galactose samples and downregulated in any of the HAT inhibitor-treated samples. The upper two figures show the relationship between genes upregulated in galactose samples and downregulated (*P* < 0.1) in any of the HAT inhibitor-treated samples. The upper left figure is based on three samples cultured in galactose-containing medium for 4 days, and the upper right figure is based on three samples cultured in galactose-containing medium for 6 days. The lower figure shows the relationship between 278 genes showing altered expression in Day 4 samples and 296 genes showing altered expression in Day 6 samples. (B) Venn diagram of genes downregulated by galactose and upregulated by treatment with any HAT inhibitor. The upper two figures show the relationship between genes downregulated by galactose and upregulated by treatment with any HAT inhibitor (*P* < 0.1). The upper left figure is based on three samples cultured in galactose-containing medium for 4 days, and the upper right figure is based on three samples cultured in galactose-containing medium for 6 days. The lower figure shows the relationship between 269 genes showing altered expression in the Day 4 samples, and 274 genes showing altered expression in the Day 6 samples.

Genes upregulated by galactose and downregulated by HAT inhibitors in the three replicates for galactose Day 4 and Day 6 were plotted in a Venn diagram (Fig 4A). A total of 278 genes were extracted from Day 4 samples and 296 genes were extracted from Day 6 samples. The total number of genes extracted from Day 4 and Day 6 samples was 421, whereas 153 genes were extracted from both Day 4 and Day 6 samples (Fig 4A). Next, genes downregulated by galactose and restored by HAT inhibitor on Day 4 and Day 6 were plotted on a Venn diagram (Fig 4B). A total of 269 genes were extracted from Day 4 samples, and 274 genes were extracted from Day 6 samples. The total number of genes extracted from Day 4 and Day 6 samples was 412, with 131 genes extracted from both Day 4 and Day 6 samples (Fig 4B). The number of genes extracted from at least one of the galactose samples increased by 421 and decreased by 412 (“union” row at *P* < 0.1; S4 Table), and the number of genes commonly extracted from all galactose samples increased by 25 and decreased by 14 (“intersection” row at *P* < 0.1; S4 Table). Genes upregulated by galactose and downregulated by HAT inhibitors overlapped more than genes that were downregulated by galactose and upregulated by HAT inhibitors.

Next, cluster analysis was performed on the 421 genes upregulated by galactose and downregulated by HAT inhibitors (S3A Fig). For this analysis, the control was placed at the farthest position. The galactose group and the galactose + HAT inhibitor groups were classified into different clusters, but the galactose group and the galactose + monotreatment group were partially mixed and clustered. Within the galactose group, five samples were clustered close together, and only Day 4 replicate 1 was located slightly farther away. Next, PCA analysis was performed (S3B Fig). As in the cluster analysis, the control was distributed farthest from the other groups. The galactose group, the HAT inhibitor group showing a cataract treatment effect, and the monotherapy group without any treatment effect were distributed axially or clustered, respectively, but the galactose group and the monotherapy group, and the treatment group and the monotherapy group, were partially mixed. The treatment group was located closer to the galactose group than to the control. C646+CPTH2 was located the farthest from the control. The positions of the galactose group and the single agents with no therapeutic effect were very close.

We next determined which genes were altered by HAT inhibitors (TH1834, C646+CPTH2, or CBP30+CPTH2) that had therapeutic effects. Genes upregulated by galactose (three samples of galactose Day 4 and three samples of galactose Day 6) and downregulated by HAT inhibitors were classified according to the number of galactose cultured samples from which they were commonly extracted and shown as a bar graph (S4A Fig). As the number of common samples increased by 1, the number of extracted genes increased about 2-fold, suggesting that, as mentioned above, there were individual differences among the galactose cultured samples, and that different genes were extracted from each replicate. As an alternative approach, we calculated the median signal value for each gene in the six galactose samples and used that signal value to similarly extract genes that were upregulated by galactose and downregulated by HAT inhibitors compared with controls. The results showed that the genes commonly extracted from the three galactose samples were similar to the genes extracted after the median was determined (S4B Fig). Therefore, we extracted 135 genes using the median value as the criterion, and proceeded with further analyses. To further refine candidate genes, we focused on samples that had no effect in monotherapy (C646, CPTH2, CBP30) but alleviated cataracts in combination treatment (C646+CPTH2, CBP30+CPTH2). Of the 43 genes that were altered in the C646+CPTH2 group, which showed a therapeutic effect, 15 genes were extracted by eliminating genes that were altered in the C646 and CPTH2 monotherapy groups, which showed no therapeutic effect (S5A Fig). Similarly, of the 71 genes extracted from the CBP30+CPTH2 group, 36 were finally extracted after excluding the genes that were also altered by monotherapy (S5B Fig). For extraction of TH1834 samples, 34 genes were extracted by eliminating genes with <2-fold variation because it was not possible to eliminate genes that varied with a single ineffective drug (S5C Fig). In total, 70 candidate genes were extracted from the treatment group (S5D Fig and S4 Dataset).

### Genes whose expression is altered by cataract therapy

**Fig 5:**
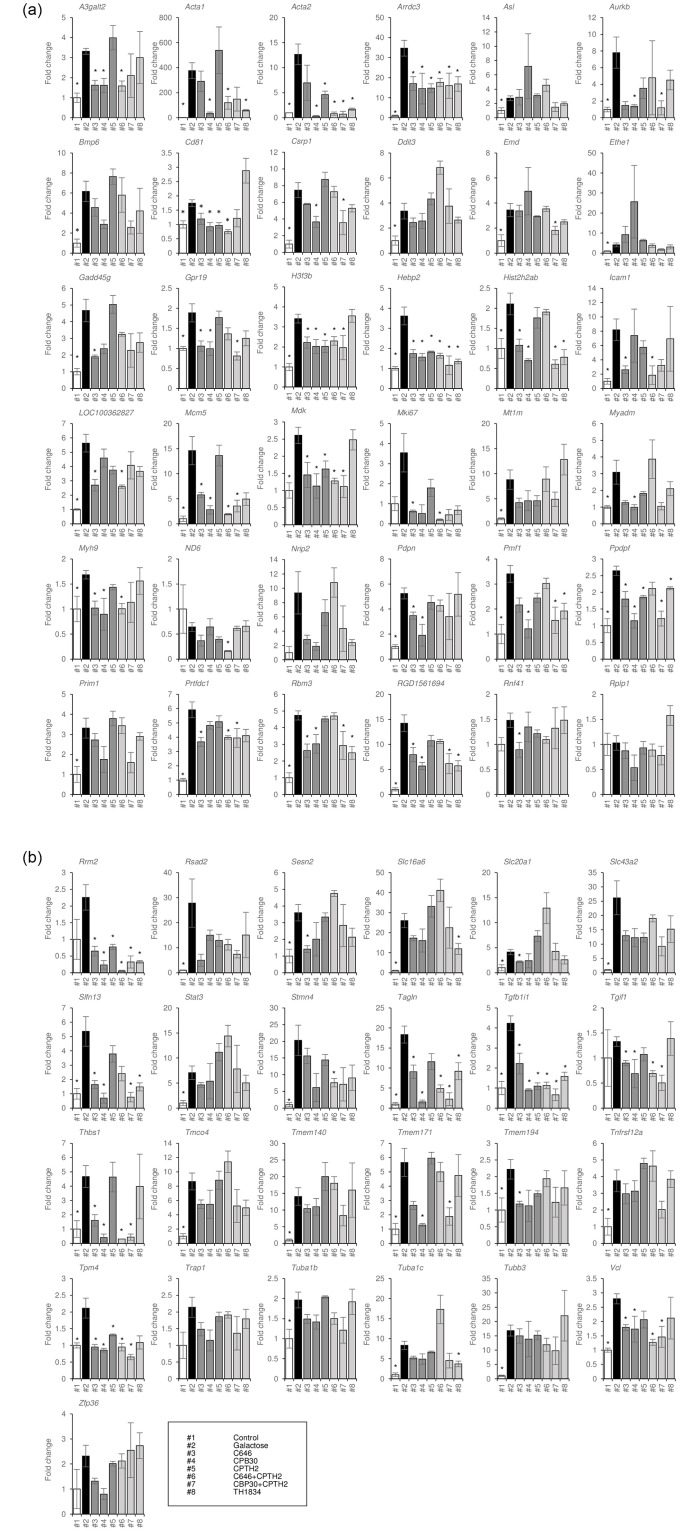
Real-time qRT-PCR results showing genes upregulated by galactose and downregulated by treatment with any HAT inhibitor. We measured expression levels of 61 genes out of 70 genes (shown in S5D Fig) for which we were able to make primers and perform real-time qRT-PCR. mRNA levels were normalized against *Gapd*h expression. Data are expressed as the mean ± SE. *P < 0.05 versus galactose. #1 control, #2 galactose, #3 C646, #4 CBP30, #5 CPTH2, #6 C646+CPTH2, #7 CBP30+CPTH2, and #8 TH1834.

**Fig 6:**
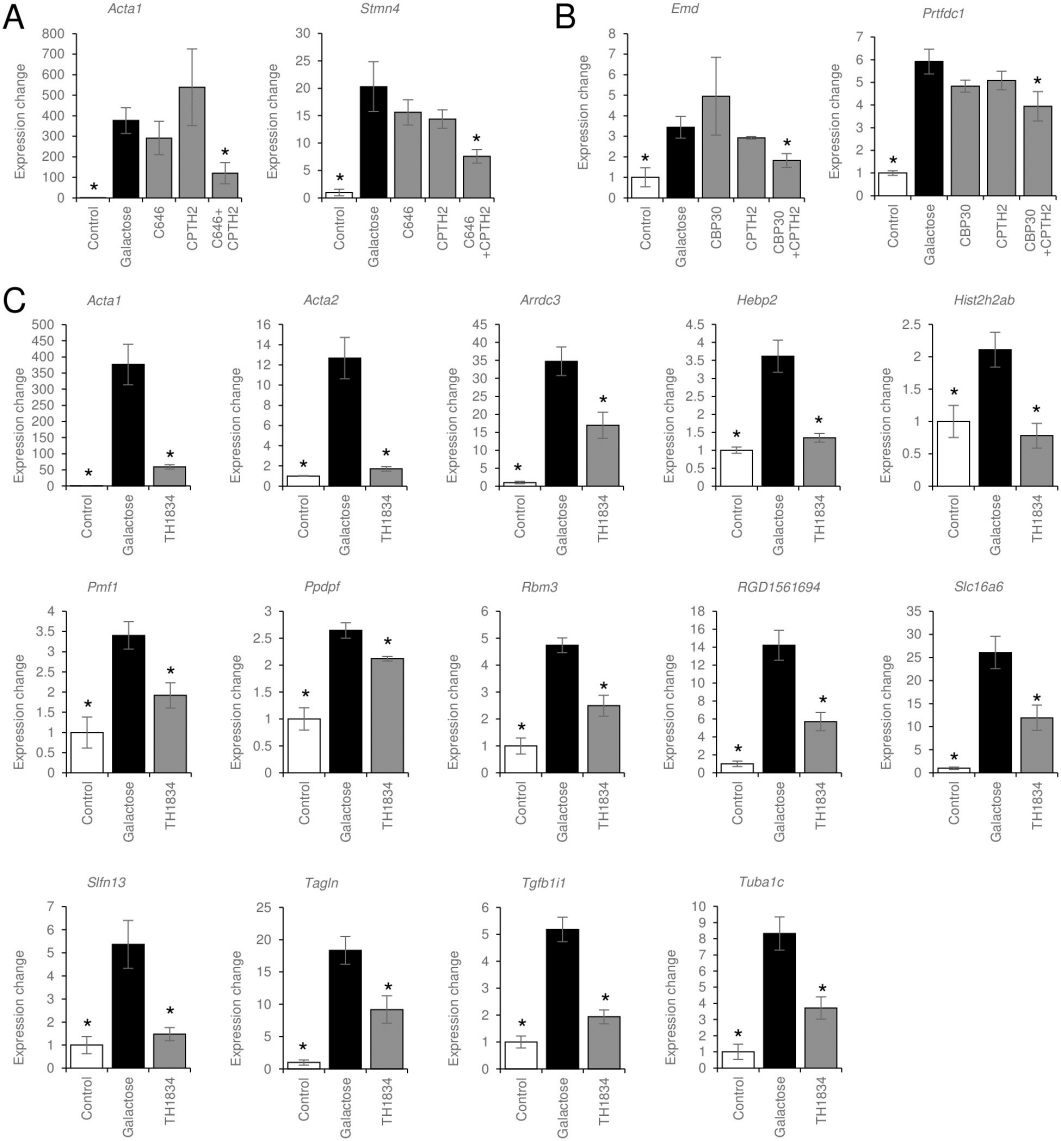
Real-time qRT-PCR of genes showing significant changes in expression in any treated sample compared with galactose alone. (A) Real-time qRT-PCR results for two out of 70 genes extracted by microarray analysis that showed significant differences in both the control and C646+CPTH2 groups compared with the galactose-only group, but no significant differences in either the C646 monotherapy or CPTH2 monotherapy groups compared with the galactose-only group. For *Acta1*, the expression level in the control sample was very low; therefore, the difference between the control and galactose-only samples may have been overestimated. (B) Real-time qRT-PCR results for two genes that showed significant differences in expression in the control and CBP30+CPTH2 groups compared with the galactose-only group, but no significant differences in either the CBP30 monotherapy or CPTH2 monotherapy groups compared with the galactose-only group. (C) Real-time qRT-PCR results for 14 genes that showed significant differences in the control and TH1834 groups compared with the galactose-only group. Target gene mRNA levels are normalized to those of *Gapdh* mRNA. Data are expressed as the mean ± SE. *P < 0.05, relative to galactose.

To confirm the results of microarray analysis, we examined the expression levels of extracted genes by real-time qRT-PCR. Among the genes for which we were able to design primers, 52 showed increased expression in the galactose group relative to the control group (Fig 5). Nineteen genes were downregulated in galactose samples treated with C646+CPTH2 compared with galactose alone. Among these, 16 downregulated by C646 monotherapy (*A3galt2*, *Arrdc3*, *Cd81*, *H3f3b*, *Hebp2*, *Icam1*, *LOC100362827*, *Mcm5*, *Mdk*, *Myh9*, *Prtfdc1*, *Tagln*, *Tgfb1i1*, *Thbs1*, *Tpm4*, and *Vcl*) and eight downregulated by CPTH2 monotherapy (*Acta2*, *Arrdc3*, *Cd81*, *H3f3b*, *Hebp2*, *Mdk*, *Tgfb1i1*, and *Tpm4*) were eliminated (Fig 5). Genes that were decreased by only C646+CPTH2 treatment were *Acta1*, a cytoskeletal protein belonging to the actin family [41], and *Stmn4*, a member of the statin family that plays a role in microtubule depolymerization, which integrates signaling pathways related to cell proliferation and differentiation [42, 43] (Fig 6A).

Among the genes whose expression was increased by galactose, 23 were downregulated in the CBP30+CPTH2 group relative to galactose alone (Fig 5). Among them, 21 that were also decreased by CBP30 monotherapy (*Acta2*, *Arrdc3*, *Aurkb*, *Csrp1*, *Gpr19*, *H3f3b*, *Hebp2*, *Hist2h2ab*, *Mcm5*, *Mdk*, *Pmf1*, *Ppdpf*, *Rbm3*, *RGD1561694*, *Slfn13*, *Tagln*, *Tgfb1i1*, *Thbs1*, *Tmem171*, *Tpm4*, and *Vcl*) and eight that were also decreased by CPTH2 monotherapy (*Acta2*, *Arrdc3*, *H3f3b*, *Hebp2*, *Mdk*, *Ppdpf*, *Tgfb1i1*, and *Tpm4*) were eliminated (Fig 5). Genes that were decreased by only CBP30+CPTH2 treatment were nuclear membrane proteins, including *Emd*, which regulates gene expression through the regulation of nuclear and cytoskeletal actin polymerization [44], and *Prtfdc1*, which inhibits cell proliferation in tumor cells [45] (Fig 6B).

Among the genes upregulated by galactose, 14 were decreased in the TH1834 group relative to galactose alone (*Acta1*, *Acta2*, *Arrdc3*, *Hebp2*, *Hist2h2ab*, *Pmf1*, *Ppdpf*, *Rbm3*, *RGD1561694*, *Slc16a6*, *Slfn13*, *Tagln*, *Tgfb1i1*, and *Tuba1c*) (Fig 6C). These genes included *Arrdc3* [46] and *Tagln* [47], which are involved in cell proliferation and apoptosis, and *Acta2* [48] and *Tgfb1i1* [49], which are involved in the epithelial-mesenchymal transition (EMT). These genes were downregulated not only in the TH1834 group, but also in the C646+CPTH2 and CBP30+CPTH2 groups, and were rescued by both drug combinations in all treated samples. We eliminated genes that were altered by ineffective monotherapies, but the combined action of these genes could still be involved in the mechanism underlying the effects of these drugs on cataracts.

## Discussion

Osmotic stress induced by a high glucose environment, apoptosis of LECs, and abnormal cell proliferation contribute to formation of diabetic cataracts [6, 7, 9, 11]. To better understand the mechanisms underlying cataract development and to prevent or treat cataracts, the effects of various drugs on cataracts have been evaluated. However, the mechanism underlying diabetic cataract development remains incompletely understood, and effective preventive and therapeutic drugs have not yet been developed.

Histone acetyltransferase (HAT) reversibly changes the conformation of histones through acetylation, thereby activating expression of previously silenced genes. By contrast, histone deacetylases repress gene expression. In the present study, we found that TH1834, which targets the HAT Tip60, was effective against galactose-induced cataract formation *ex vivo* and reduced opacity in most of the cortical equatorial region (Fig 1D). CPTH2, a Gcn5 inhibitor, had a weak therapeutic effect on cataracts, and decreased opacity slightly (Figs 1D and 2C and S1 Fig). Our prior study identified cataract-preventive effects of 16 differently targeted HAT inhibitors, suggesting that each inhibits cataract formation via several different pathways [15]. Therefore, we tested the possibility of reversing cataract formation by combined treatment with inhibitors targeting distinct HATs, and found that the combination of C646 and CPTH2 (a CBP/p300 inhibitor and a Gcn5 inhibitor) had a therapeutic effect (Fig 2A and 2C and S1 Fig). The combination of CBP30, which targets CBP/p300, and CPTH2 also had a therapeutic effect (Fig 2A and 2C and S2 Fig). It is possible that the weak therapeutic effect of CPTH2 was augmented by C646 and CBP30, resulting in a significant therapeutic effect. When sections of the lens were prepared and stained with H&E, vacuoles were observed in the cortical equatorial region in the galactose-only group, and the tissue was greatly disorganized, whereas few vacuoles were observed in treated samples. In addition, treated samples exhibited traces of crushed vacuoles, and nucleus-containing cells were present in the equatorial transition zone (Fig 2B). This suggests that the thinning of the ring-shaped opacity reflects normalization of the collapsed cortical fibers. Punctate structures remained in the sample treated with TH1834, but this may be part of the crushed blanks. Cortical opacities with a punctate and zonal shape are a characteristic morphological feature of galactose-induced cataracts (Fig 1D). Meydani et al. reported a representative cataract grading system [40]. In short, a cataractous lens shows small subcapsular and/or outer cortical vacuoles. Cortical vacuoles progress toward the inner cortex, and then large opaque areas are seen against a transparent background.

In the present study, we investigated genes whose expression is altered by cataract treatment. To do this, we performed a comprehensive analysis of gene expression levels using microarray analysis. First, to identify genes related to cataract formation, we compared expression changes in non-cataractous lenses (control), early cataracts (lenses cultured in galactose for 4 days), and post-cataracts (lenses cultured in galactose for 6 days). Most of the 933 genes were upregulated maximally after 4 days of incubation, and no change in expression was observed after Day 4 (S3 Table). However, *Hsa1b* expression increased on Day 4 and further increased on Day 6 (S3 Table). *Hsa1b* is a member of the HSP70 family of molecular chaperones that protect cells from the stress of misfolded proteins [50]. Because endoplasmic reticulum stress occurs in diabetic cataract, this gene could have been induced by the stress caused by galactose treatment [51]. In addition, expression of nine genes increased only at the early cataract stage, and that of nine genes increased only at the late cataract stage. These are putative marker genes for progression of diabetic cataract, and further investigation of their role is warranted.

To investigate genes involved in cataract treatment, we extracted 421 genes that were upregulated by galactose and decreased by treatment with any of the HAT inhibitors (S2 Dataset), and 412 genes that were decreased by galactose and increased by HAT inhibitors (S3 Dataset). These genes could potentially be involved in cataract treatment. To further select likely regulatory genes, we eliminated genes that were upregulated by galactose, decreased in the therapeutically effective HAT treatment groups, and similarly decreased by single HAT inhibitors without therapeutic effects. Because the mechanisms of action of C646+CPTH2 and CBP30+CPTH2 are not necessarily identical, we eliminated genes whose expression was altered by each single agent alone. Thus, 70 candidate genes were finally extracted (S5 Fig). Validation of this gene set by RT-qPCR revealed that expression of *Stmn4* and *Acta1* was altered predominantly in the C646+CPTH2 group; that of *Emd* and *Prtfdc1* was altered predominantly in the CBP30+CPTH2 group; and that of *Acta1*, *Acta2*, *Arrdc3*, *Hebp2*, *Hist2h2ab*, *Pmf1*, *Ppdpf*, *Rbm3*, *RGD1561694*, *Slc16a6*, *Slfn13*, *Tagln*, *Tgfb1i1*, and *Tuba1*c was altered predominantly in the TH1834 group.

Arrestin domain-containing 3 (*Arrdc3*) encodes a member of the arrestin family of proteins involved in regulation of G protein signal transduction [52]. Arrdc3 has been well-studied in breast cancer, and it inhibits proliferation of human breast cancer cells [46]. Furthermore, ARRDC3 overexpression increases apoptosis in breast cancer cells, whereas inhibition significantly inhibits apoptosis [53]. In addition, ARRDC3 is epigenetically regulated in breast cancer cells. Deacetylation of histones by class 3 HDACs results in ARRDC3 silencing [54]. The function of ARRDC3 in the lens is unknown, but it is significantly upregulated by galactose, and its expression was reduced by HAT inhibitor-mediated histone deacetylation. Tagln is involved in cell proliferation and apoptosis, and its overexpression in colorectal cancer cells inhibits cell proliferation and increases apoptosis [47]. Because apoptosis and dysregulation of cell proliferation are involved in diabetic cataract [9, 11], targeting genes that activate cell proliferation and suppress apoptosis could be an important cataract treatment; however, further studies are needed to clarify these relationships.

EMT is an abnormal morphological transcriptional programming of LECs that results in acquisition of invasive potential and resistance to apoptosis, and abnormal activation of cell proliferation [55]. EMT is involved in the pathogenesis of diabetic cataract [56]. *Acta2* (also known as α-SMA) is an actin protein and well-known marker of mesenchymal cells induced by EMT [48]. Under high glucose conditions, α-SMA is upregulated in LECs, thereby inducing EMT [57]. LEC EMT has been studied in detail in posterior cataracts after cataract surgery, revealing that expression of α-SMA is induced by TGFβ signaling in this context [58]. TGFβ-induced cataract in LECs can be prevented by HDAC inhibitors [59], which is contrary to the results of our study that HAT inhibitors can treat cataracts. However, histone acetylation in the *Acta2* promoter region is increased by TGFβ-induced EMT in cultured human lens cells, while histone acetylation is suppressed by HDAC inhibitors [60]. This suggests that HDAC inhibition regulates α-SMA through a specific factor rather than directly, and that the use of HAT inhibitors in our study could have suppressed α-SMA expression through a different pathway. Although our microarray results did not reveal increased expression of TGFβ (-1, -2, -3), which induces α-SMA expression, high expression of TGFb1I1 induces EMT in some cell types [49]. The role of EMT in cortical cataracts is unknown, but galactose increased expression of EMT factors, which was then suppressed by HAT inhibitors. These results suggest that normal proliferation of normal lens epithelial cells, which is regulated by a balance between survival and cell death, could be important for the treatment of cataracts. For this extraction method, the final extracted genes were not common to all three cataract treatment samples. However, *Arrdc3*, *Tagln*, *Acta2*, and *Tgfb1i1* were downregulated not only in the TH1834 group but also in the C646+CPTH2 and CBP30+CPTH2 groups, suggesting that the combined action of these genes could be involved in the mechanism underlying cataract treatment effects.

In conclusion, we demonstrated that HAT inhibitors treat cataracts by altering expression of target genes primarily related to cell proliferation and differentiation. We suggest that HAT inhibitors and their downstream factors represent a new putative approach to treating diabetic cataracts. This study was limited to investigating HAT regulation of gene expression levels, and further investigation of changes in the functional status of lens cells, such as apoptosis levels and cell proliferative capacity, will be needed to further elucidate the mechanism underlying cataract treatment.

## Supporting information

S1 Fig Effect of HAT inhibitors on the galactose-induced cataracts used in this study. The upper part of the photograph shows an image taken before addition of the inhibitor, and the lower part shows an image taken after addition of the inhibitor. In the photograph, “q” on the left denotes the sample used for qRT-PCR, and “M” denotes the sample used for microarray analysis.
(PDF)

S2 Fig Photograph of galactose-treated samples used for microarray analysis. The top three photographs show galactose samples from Day 4, and the bottom three photographs show galactose samples from Day 6.
(PDF)

S3 Fig Microarray analysis identified genes whose expression is altered by HAT inhibitors. Microarray analysis was performed on 6 samples: lenses cultured with HAT inhibitors (C646, CBP30, CPTH2, C646+CPTH2, CBP30+CPTH2, TH1834) after cataract induction with galactose (n = 1 for each HAT inhibitor and HAT inhibitor combination). (A) Heatmap of genes downregulated in HAT inhibitor-treated samples. The red to green gradient indicates the weight of the signal, with higher values in red and lower values in green. (B) PCA plots of 421 genes (shown in Fig 3A) upregulated in galactose samples and downregulated in any of the HAT inhibitor-treated samples. The closer samples are to one another on the plot, the higher the expression profile homology.
(PDF)

S4 Fig The differences in the extracted expression variation between the two different analysis methods. (A) Genes differentially expressed between the control sample and any of the six galactose samples were identified, and the number of genes altered in each number of samples was plotted on a bar graph. (B) Upper left, Venn diagram based on the median values of the six galactose samples. The Venn diagram shows the relationship between the number of genes whose expression increased in galactose samples compared with control samples and decreased after treatment. Upper right, Venn diagram of genes with variable expression that overlap in three of the six samples extracted based on individual galactose samples. The Venn diagram shows the relationship between the number of genes showing increase expression in three galactose replicates compared with control and decreased expression after treatment. Bottom center, relationship between genes selected by median analysis and genes that fluctuated in all three galactose replicates.
(PDF)

S5 Fig Extraction of genes whose expression was increased by galactose and repressed by HAT inhibitor treatment. (A) Venn diagram showing the relationship between the 42 genes that were increased by galactose (median = six galactose samples) and downregulated by C646+CPTH2, and genes that were downregulated by C646 or CPTH2. Fifteen genes were decreased only by C646+CPTH2. (B) Venn diagram showing the relationship between the 71 genes increased by galactose and decreased by CBP30+CPTH2, and genes that were decreased by CBP30 or CPTH2. Thirty-six genes were decreased only by CBP30+CPTH2. (C) Genes upregulated by galactose and downregulated by TH1834. Among the genes selected using the criterion *P* < 0.1, 34 genes were downregulated >2-fold in TH1834-treated sample compared with galactose-only samples. (D) Venn diagram showing the relationship between the 70 genes showing altered expression in the three HAT inhibitors (C646+CPTH2, CBP30+CPTH2, TH1834) treatment samples.
(PDF)

S1 Table List of primers used for real-time qRT-PCR. (DOCX)

S2 Table List of HAT inhibitors. In the "Therapeutic effect" column, "○" indicates that the HAT inhibitor alone had a complete therapeutic effect, "Δ" indicates that the HAT inhibitor alone had a slight therapeutic effect, and "×" indicates that the HAT inhibitor alone had no therapeutic effect.
(DOCX)

S3 Table Genes differentially expressed over time during galactose incubation. The column "ctrl-Galday4" indicates whether gene expression increased or decreased relative to that in the control after 4 days of incubation with galactose. The "Galday4-Day6" column shows whether gene expression increased or decreased from Day 4 to Day 6 of incubation with galactose.
(DOCX)

S4 Table Number of genes whose expression increased or decreased after exposure to galactose, and decreased or increased after addition of the HAT inhibitor. This table shows the number of genes whose expression in each galactose sample increased or decreased relative to that in the control sample, and was decreased or increased by treatment with any one of three therapeutic agents. Significance was defined as *P* < 0.1, *P* < 0.05, and *P* < 0.01, and the number of each was noted. The same analysis was repeated for six galactose samples. The “Union” row shows the number of genes selected from any of the six galactose samples. The “Intersect” line shows the number of genes selected from all six galactose samples.
(DOCX)

S1 Dataset List of 1483 genes that were significantly (*P* < 0.1) increased or decreased in any galactose-treated sample. Genes whose expression level was significantly higher (*P* < 0.1) in galactose than in the control are marked "UP," and those whose expression level was significantly lower (*P* < 0.1) in galactose than in the control are marked "DOWN." The six columns on the right show the change (Log2) in the signal value for each galactose sample relative to the control sample.
(XLSX)

S2 Dataset List of 421 genes whose expression is increased by galactose and decreased by any of the HAT inhibitors. In each galactose sample, genes that correspond to the conditions are indicated by “●”. Thirteen columns on the right side show the signal value for each gene. This gene list does not show changes in expression levels, only the signal values themselves.
(XLSX)

S3 Dataset List of 412 genes whose expression is decreased by galactose and increased by any of the HAT inhibitors. In each galactose sample, genes that correspond to the conditions are indicated by "●." The 13 columns on the right side show the signal value for each gene.
(XLSX)

S4 Dataset List of 70 genes whose expression levels changed in the three treatment samples. Genes (shown in Fig 4) whose expression levels changed significantly in each treatment sample are indicated by "◆". The difference between the signal value for each treatment sample and the median signal value for the six galactose samples is shown in the seven columns on the right.
(XLSX)
