# Supplementary figures and images for: Histone acetyltransferase inhibition reverses opacity in rat galactose-induced cataract
Source: PLoS One. 2022 Nov 23;17(11):e0273868. doi: 10.1371/journal.pone.0273868 (PMC9683626; doi:10.1371/journal.pone.0273868)

## Galactose (n=8)

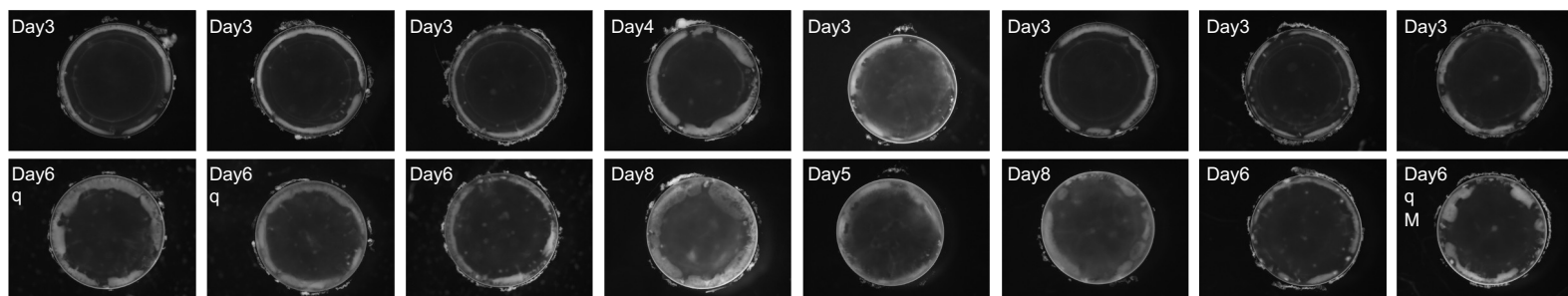

## C646 (n=3)

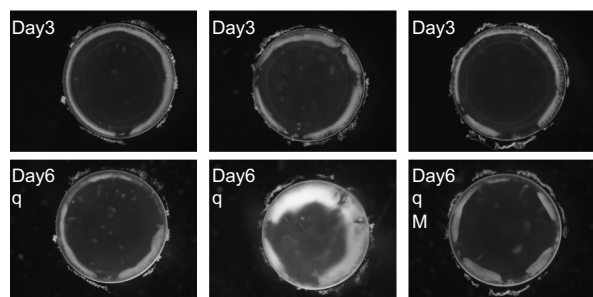

## CBP30 (n=3)

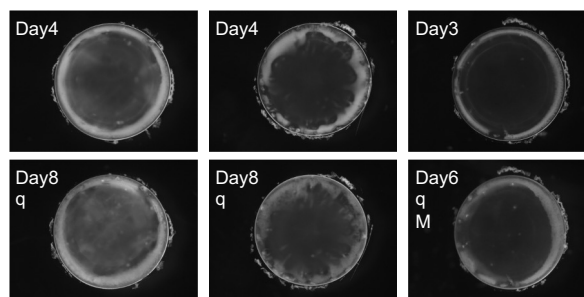

## CPTH2 (n=3)

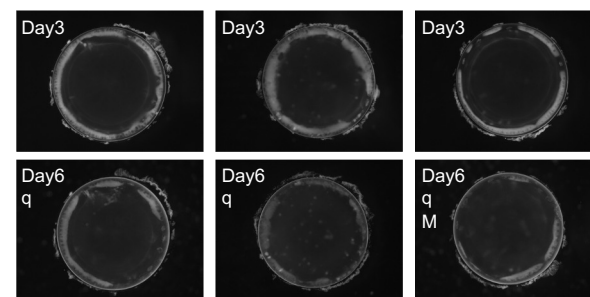

## C646+CPTH2 (n=3)

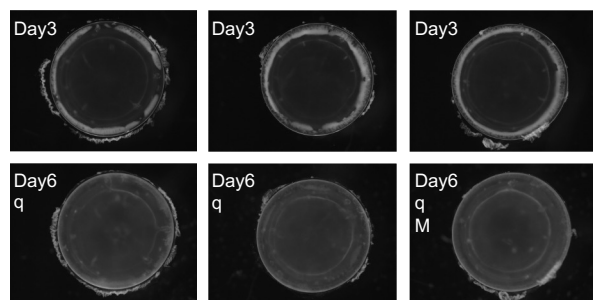

## CBP30+CPTH2 (n=3)

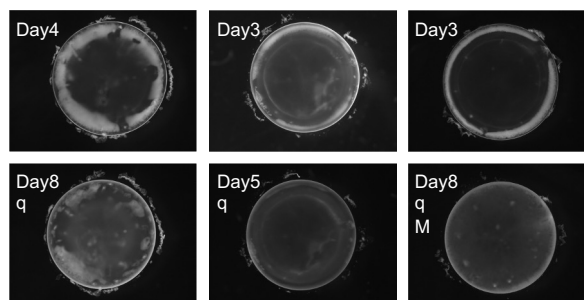

## TH1834 (n=3)

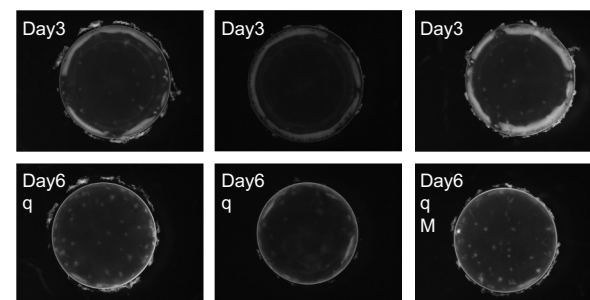

Supplement: S1 Fig — The upper part of the photograph shows an image taken before addition of the inhibitor, and the lower part shows an image taken after addition of the inhibitor. In the photograph, “q” on the left denotes the sample used for qRT-PCR, and “M” denotes the sample used for microarray analysis. (PDF) [file pone.0273868.s001.pdf]

Galactose Day4-1

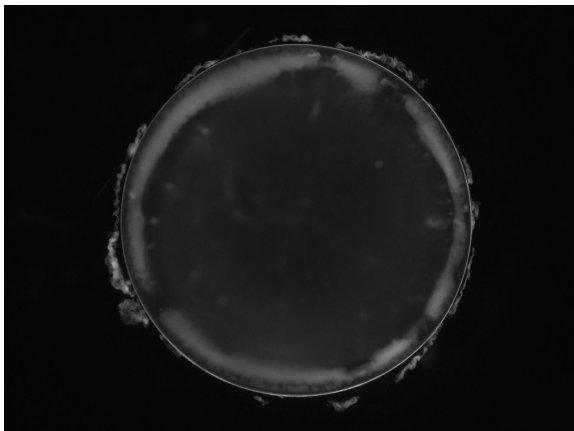

Galactose Day4-2

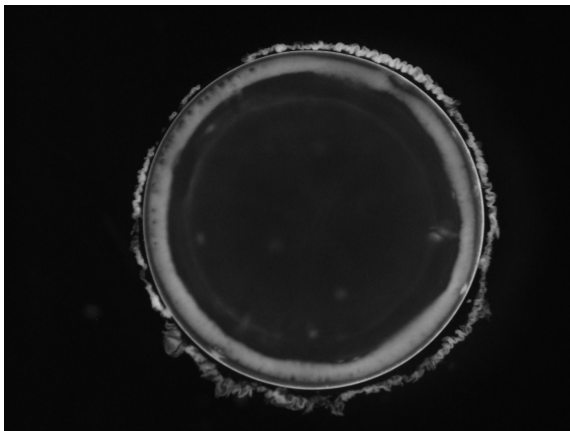

Galactose Day4-3

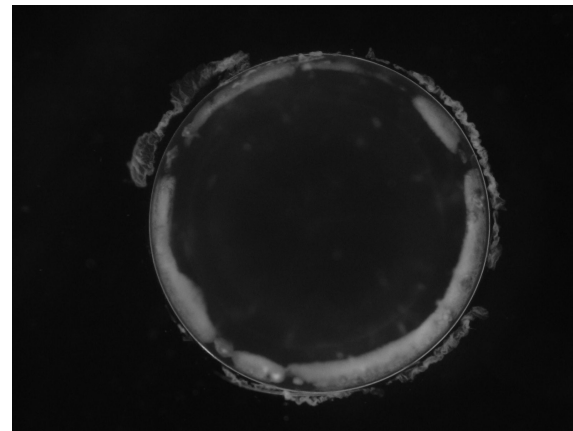

Galactose Day6-1

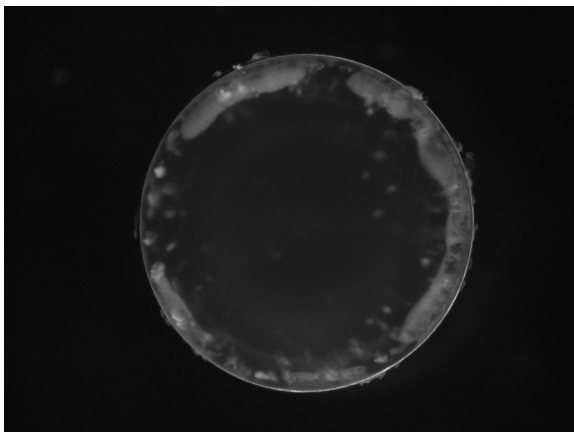

Galactose Day6-2

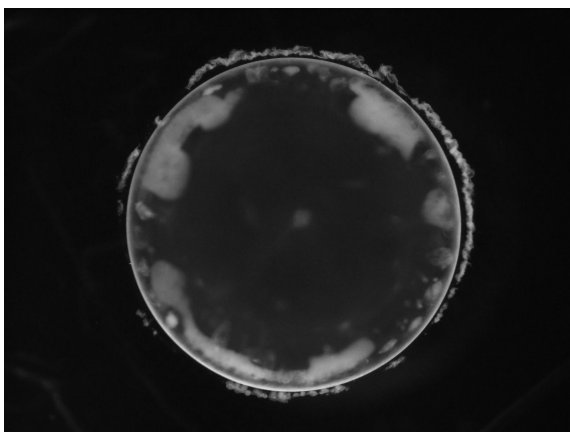

Galactose Day6-3

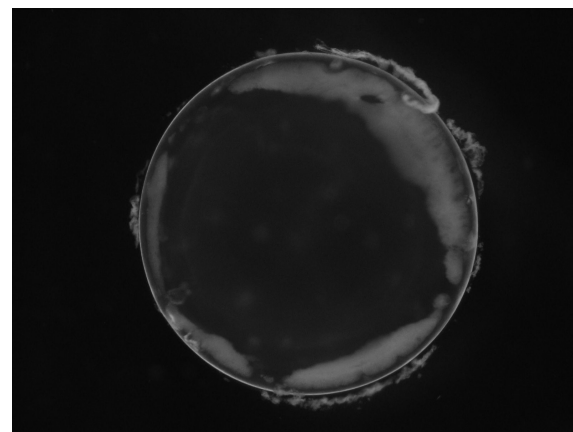

Supplement: S2 Fig — The top three photographs show galactose samples from Day 4, and the bottom three photographs show galactose samples from Day 6. (PDF) [file pone.0273868.s002.pdf]

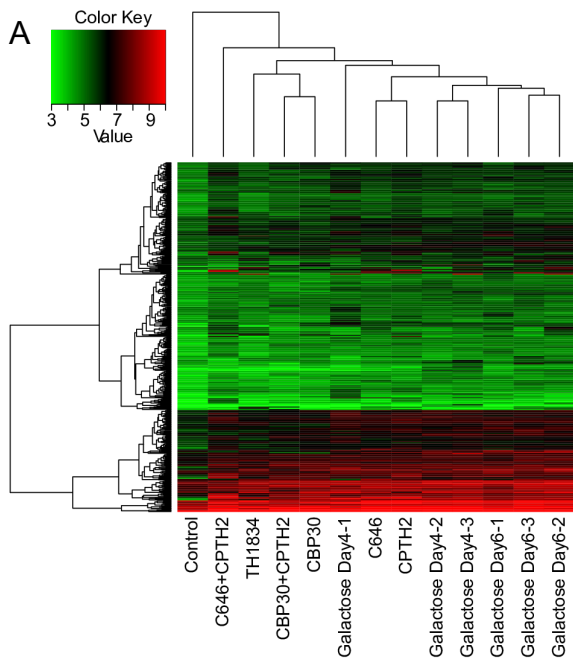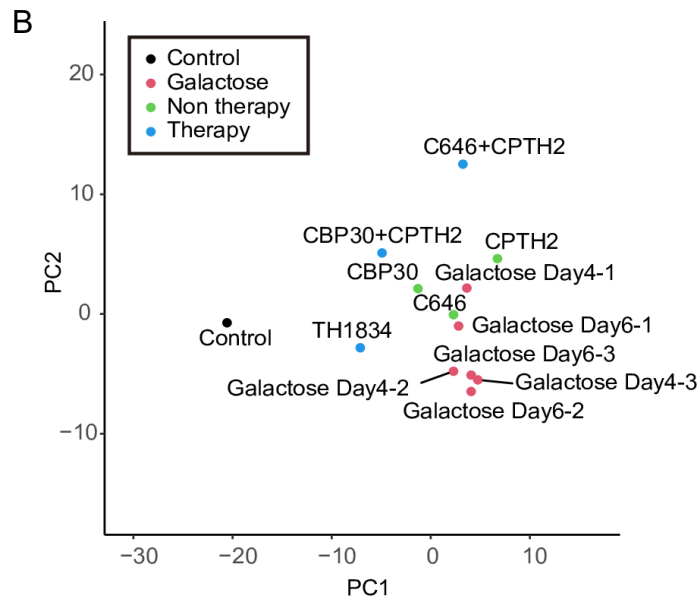

Supplement: S3 Fig — Microarray analysis was performed on 6 samples: lenses cultured with HAT inhibitors (C646, CBP30, CPTH2, C646+CPTH2, CBP30+CPTH2, TH1834) after cataract induction with galactose (n = 1 for each HAT inhibitor and HAT inhibitor combination). (A) Heatmap of genes downregulated in HAT inhibitor-treated samples. The red to green gradient indicates the weight of the signal, with higher values in red and lower values in green. (B) PCA plots of 421 genes (shown in Fig 3A) upregulated in galactose samples and downregulated in any of the HAT inhibitor-treated samples. The closer samples are to one another on the plot, the higher the expression profile homology. (PDF) [file pone.0273868.s003.pdf]

A

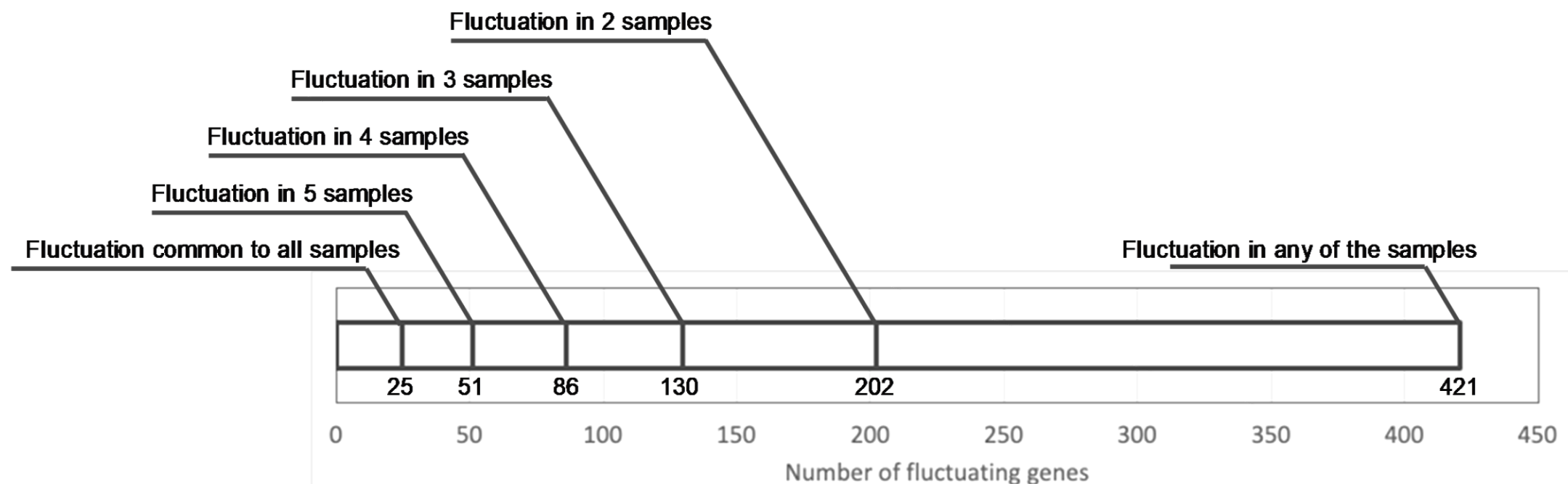

B

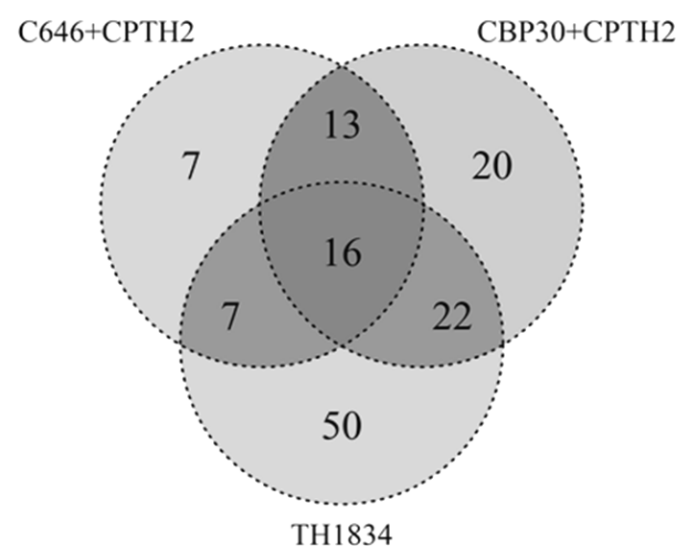

Mediam(135)

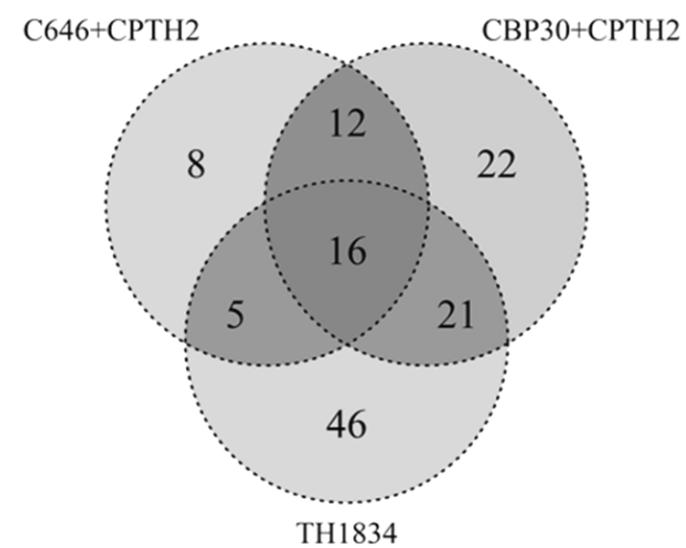

Common 3 Samples  
(130)

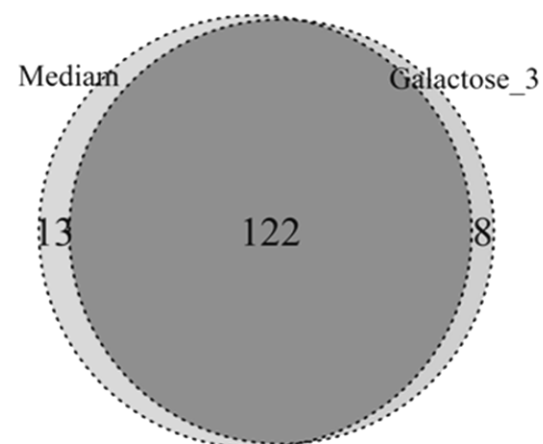

Supplement: S4 Fig — (A) Genes differentially expressed between the control sample and any of the six galactose samples were identified, and the number of genes altered in each number of samples was plotted on a bar graph. (B) Upper left, Venn diagram based on the median values of the six galactose samples. The Venn diagram shows the relationship between the number of genes whose expression increased in galactose samples compared with control samples and decreased after treatment. Upper right, Venn diagram of genes with variable expression that overlap in three of the six samples extracted based on individual galactose samples. The Venn diagram shows the relationship between the number of genes showing increase expression in three galactose replicates compared with control and decreased expression after treatment. Bottom center, relationship between genes selected by median analysis and genes that fluctuated in all three galactose replicates. (PDF) [file pone.0273868.s004.pdf]

**A**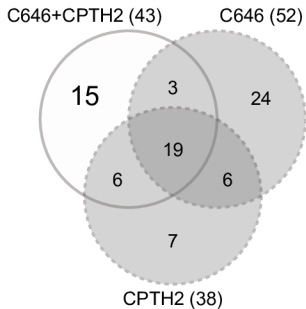**B**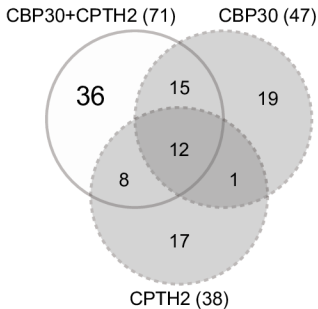**C**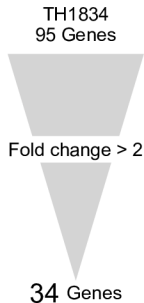**D**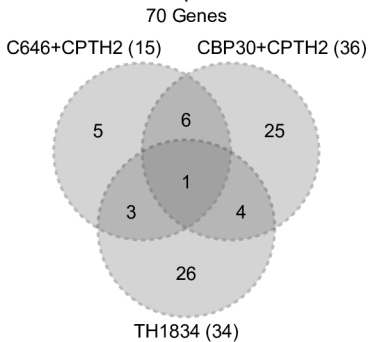

Supplement: S5 Fig — (A) Venn diagram showing the relationship between the 42 genes that were increased by galactose (median = six galactose samples) and downregulated by C646+CPTH2, and genes that were downregulated by C646 or CPTH2. Fifteen genes were decreased only by C646+CPTH2. (B) Venn diagram showing the relationship between the 71 genes increased by galactose and decreased by CBP30+CPTH2, and genes that were decreased by CBP30 or CPTH2. Thirty-six genes were decreased only by CBP30+CPTH2. (C) Genes upregulated by galactose and downregulated by TH1834. Among the genes selected using the criterion P < 0.1, 34 genes were downregulated >2-fold in TH1834-treated sample compared with galactose-only samples. (D) Venn diagram showing the relationship between the 70 genes showing altered expression in the three HAT inhibitors (C646+CPTH2, CBP30+CPTH2, TH1834) treatment samples. (PDF) [file pone.0273868.s005.pdf]
