# Supplementary material for: Histone acetyltransferase inhibition reverses opacity in rat galactose-induced cataract
Source: PLoS One. 2022 Nov 23;17(11):e0273868. doi: 10.1371/journal.pone.0273868 (PMC9683626; doi:10.1371/journal.pone.0273868)
Supplement: S1 Table — (DOCX) [file pone.0273868.s006.docx]

| Gene | Forward Primer | Reverse Primer |
| --- | --- | --- |
| *A3galt2* | 5'-GGGACTCAGGGCCAAGAAG-3' | 5'-CAGGCCTAGGAGACCAAACG-3' |
| *Acta1* | 5'-TCAGGCGGTGCTGTCTCTCT-3' | 5'-TCCCCAGAATCCAACACGAT-3' |
| *Acta2* | 5'-ACCAGGGAGTGATGGTTGGA-3' | 5'-CGCTTCGTCCCCCACATA-3' |
| *Arrdc3* | 5'-CGGTCTATCCCTTCCTGAAAGA-3' | 5'-TCCTCCGTTACAACCTCTGCAT-3' |
| *Asl* | 5'-CAGGCAGAAGTCGCAATGAC-3' | 5'-GAGCAGGTTTGCCTCATCCA-3' |
| *Aurkb* | 5'-GACTTTGGCTGGTCTGTGCAT-3' | 5'-GGTGCCGCACATGGTCTT-3' |
| *Bmp6* | 5'-CGCTCCGCTCTTCATGCT-3' | 5'-TGACACCCCATCCTCTTCGT-3' |
| *Cd81* | 5'-TGCCAAGGCAGTGGTGAAG-3' | 5'-GCGTATTGGAGCCACAACAGT-3' |
| *Csrp1* | 5'-CCAGTGCGAGGGCAACA-3' | 5'-TGCAAACCATGCACAGGAA-3' |
| *Ddit3* | 5'-TCATCCCCAGGAAACGAAGA-3' | 5'-AGCTAGGGATGCAGGGTCAA-3' |
| *Emd* | 5'-CCCAGCTCGTCATCGTCTTC-3' | 5'-CCACGGAGGCTGAATCCA-3' |
| *Ethe1* | 5'-CGCGAAGACTTTGTACCACTCA-3' | 5'-AGACAGTTGCCTGGAAGTGTGA-3' |
| *Gadd45g* | 5'-GAGTCCGCCAAAGTCCTGAA-3' | 5'-AGCCAGCACGCAAAAGGT-3' |
| *Gapdh* | 5'-CGTATCGGACGCCTGGTTA-3' | 5'-GATGGCAACAATGTCCACTTTG-3' |
| *Gpr19* | 5'-GCCCCCTGTGGTTACTGCTA-3' | 5'-CTTCCACACAGCTGCCATTCT-3' |
| *H3f3b* | 5'-AGGGCCTCAGACTTCAGCTTT-3' | 5'-TCCTAGCGGTCTGCTTGGTT-3' |
| *Hebp2* | 5'-CCGCCTTGTGGCTCAAAC-3' | 5'-ACGCACGGCCCTGAAA-3' |
| *Hist2h2ab* | 5'-CAACAAGAAGACCCGCATCA-3' | 5'-AGCTCTTCATCGTTCCTTACAGCTA-3' |
| *Icam1* | 5'-ATTGCGGGCTTCGTGATC-3' | 5'-GATCTTCCTCTGGCGGTAATAGG-3' |
| *LOC100362827* | 5'-GAAGGACGAGCCACAGAGAAGA-3' | 5'-GCTTCGGAGGAGCAGGTTT-3' |
| *Mcm5* | 5'-GTGAGATGCCCAGGCACAT-3' | 5'-CCAGGAACAACCTTGTCACACA-3' |
| *Mdk* | 5'-GGTGCCCTGCAACTGGAA-3' | 5'-CCCCAGCTCTCAAACTTGTATTTG-3' |
| *Mki67* | 5'-ATTTCAGTTCCGCCAATCC-3' | 5'-GGCTTCCGTCTTCATACCTAAA-3' |
| *Mt1m* | 5'-GCTCCTAGAACTCTACAGCGATCTC-3' | 5'-GGGTCCATGGCGAATGG-3' |
| *Myadm* | 5'-TTCGCAGCCGGAACTTTC-3' | 5'-CGGCATGGCTGCTTCCT-3' |
| *Myh9* | 5'-CAGAGACTGCCGATGCTATGAA-3' | 5'-TGTCCCCACGCCTCAGTT-3' |
| *ND6* | 5'-TGTCTAGGGTTGGCGTTGAAG-3' | 5'-CCAATACATCCACTAACAATCAATCC-3' |
| *Nrip2* | 5'-CAGGTTCCAGCTCTTCTTGTCA-3' | 5'-CACAGCCACACGAAGCATCT-3' |
| *Pdpn* | 5'-CCACGGACAAGAAAACAACTCA-3' | 5'-TCTTATCTGTGGTCTGCGTTTCA-3' |
| *Pmf1* | 5'-AGTCGCGGGCAAGAGCTA-3' | 5'-GGGTTCAACTGGTGCAAGTGT-3' |
| *Ppdpf* | 5'-GGAAATCCACCCTCCCATTC-3' | 5'-TGCCGAGTGCTCAGGAGACT-3' |
| *Prim1* | 5'-AATGGCCATGCGCATCATA-3' | 5'-GGCGGTGCTTAAATCCAAAG-3' |
| *Prtfdc1* | 5'-CAGCAGCATTGAGAAATACAAACC-3' | 5'-CGCTGTCCTCTTCACCAATAAA-3' |
| *Rbm3* | 5'-GCAGCTTCGGGCCTATCTCT-3' | 5'-ACCCCGGGATCTTTGAGTCT-3' |
| *RGD1561694*/ *RGD1559962/Hmgb2* | 5'-CGCCGAGTTCTCGAAGAAAT-3' | 5'-TCGACTTTTCCTTGGCAGACA-3' |
| *Rplp1/* *LOC100360522* | 5'-TAAGGCCGCGTTGAGGTG-3' | 5'-GATCTTATCCTCCGTGACCGT-3' |
| *Rnf41* | 5'-ACCTGCCCCCACCACTTAG-3' | 5'-TTACATCATACCCCATGTCTCATCA-3' |
| *Rrm2/* *LOC100359539* | 5'-AGCTGAGGCCTCCTTTTGG-3' | 5'-AGTGCTGAATATCCTTGGAAAGGT-3' |
| *Rsad2* | 5'-GCTGGCTGAGAATAGCATTGG-3' | 5'-ACCCGTGGCTGTCCCTTT-3' |
| *Sesn2* | 5'-TTTCGTGCCCAGGATTATACCT-3' | 5'-GGGTAGAGCCGCTGGATCA-3' |
| *Slc16a6* | 5'-CCGCGCTGCATTCTTACTGT-3' | 5'-CCGATCCTCCCGAACACTT-3' |
| *Slc20a1* | 5'-CCGTCAGCAACCAGATCAACTC-3' | 5'-CCCATGCAGTCTCCCACCTTG-3' |
| *Slc43a2* | 5'-CCACTGTTTCTGGCCATGATG-3' | 5'-GACAAGCAGACCCACGTTCA-3' |
| *Slfn13* | 5'-GCTGCAGGCCCTTGTGATT-3' | 5'-CCAAGCTGGTCGCTCAAGA-3' |
| *Stat3* | 5'-AGCTCTTAGGGCCTGGTGTGAACTACT-3' | 5'-GGATGGCCCTCTCCCGCTCCTTGCTGA-3' |
| *Stmn4* | 5'-ACCAGATTCCAGCCAACATGACCCT-3' | 5'-ACTGTCTCCCACACCAGCCTTCATA-3' |
| *Tagln* | 5'-TGGAGTGGATTGTAATGCAGTGT-3' | 5'-CCAGGCGCCCACGAT-3' |
| *Tgfb1i1* | 5'-CCCCACGATGTGGCTTCT-3' | 5'-CCAAGGCGGTGACCATTTTAT-3' |
| *Tgif1* | 5'-AGCAGACACACCTGTCCACACT-3' | 5'-TGCGACGGGCGTTGA-3' |
| *Thbs1* | 5'-AACGTGGATCAGAGGGACAC-3' | 5'-GTCATCGTCATGGTCACAGG-3' |
| *Tmco4* | 5'-CCACCGGTCTGCTGAGGTT-3' | 5'-CCCTGTTGCCTCGAGAGAAG-3' |
| *Tmem140* | 5'-AAGAACCACTGGGAGAACTGCTA-3' | 5'-TGAACCCTTGCCATCCCATA-3' |
| *Tmem171* | 5'-GGAGCAGTGAGGAGGCCTTT-3' | 5'-TCTGGCTCAGCAGTTCCTACAG-3' |
| *Tmem194* | 5'-TCCCAAAGTGGCATGATATATGG-3' | 5'-ACACGGACCAATTTGGAGCTA-3' |
| *Tnfrsf12a* | 5'-CAGCACCTCCTGCCCACTT-3' | 5'-GACTAAGAGCGCCTCCCAGAA-3' |
| *Tpm4* | 5'-GAACGGCGCGAGAAAGC-3' | 5'-TCTTCCTCCACCAGCTGGAT-3' |
| *Trap1* | 5'-GTCCAACCTTGGCACAATCG-3' | 5'-GCTGCAGTGCTTCCAGGAA-3' |
| *Tuba1b* | 5'-GGGACCCGGTGTCTGCTT-3' | 5'-GATGCACTCACGCATGATAGCT-3' |
| *Tuba1c* | 5'-TTTCGCGGACCACTTCAAG-3' | 5'-TGGCCAACGTGGATGGA-3' |
| *Tubb3* | 5'-GGGCCTTTGGACACCTATTCA-3' | 5'-CCCTTTGGCCCAGTTGTTG-3' |
| *Vcl* | 5'-TCCTGCGCGGGATTACC-3' | 5'-CAGACGTTCCAGAGAGGATTCC-3' |
| *Zfp36* | 5'-TCGCGCCACCATGGAT-3' | 5'-GTCATGGCTCATCGACATAAGG-3' |
