# Supplementary material for: Histone acetyltransferase inhibition reverses opacity in rat galactose-induced cataract
Source: PLoS One. 2022 Nov 23;17(11):e0273868. doi: 10.1371/journal.pone.0273868 (PMC9683626; doi:10.1371/journal.pone.0273868)
Supplement: S2 Table — In the "Therapeutic effect" column, "○" indicates that the HAT inhibitor alone had a complete therapeutic effect, "Δ" indicates that the HAT inhibitor alone had a slight therapeutic effect, and "×" indicates that the HAT inhibitor alone had no therapeutic effect. (DOCX) [file pone.0273868.s007.docx]

| **Name** | **Target** | **Place of purchase** | **Concentration** | **Therapeutic effect** |
| --- | --- | --- | --- | --- |
| CTK7A | p300/PCAF | EMD Millipore (USA) | 100 μM | × |
| Garcinol | p300/PCAF | Abcam (UK) | 2 μM | × |
| Anacardic Acid | p300/PCAF | Abcam (UK) | 20 μM | × |
| MG149 | TIP60/MOZ | Selleck Chemicals (USA) | 50 μM | × |
| C646 | p300 | Sigma Aldrich (USA) | 40 μM | × |
| CPTH2 | GCN5 | Cayman Chemical (USA) | 80 μM | △ |
| gallic acid | HAT | Sigma Aldrich (USA) | 100 μM | × |
| (-)-Epigallocatechin gallate (ECGC) | HAT | Wako (Japan) | 50 μM | × |
| EML425 | p300/CBP | Tocris (UK) | 200 μM | × |
| ISOX DUAL | CBP/BRD4 | Sigma Aldrich (USA) | 20 μM | × |
| Plumbagin | p300 | Sigma Aldrich (USA) | 2 μM | × |
| TH1834 | TIP60 | Axon Medchem (Netherlands) | 50 μM | ○ |
| windorphen | HAT | Sigma Aldrich (USA) | 40 μM | × |
| Remodelin | NAT10 | Cayman Chemical (USA) | 40 μM | × |
| Embelin | PCAF | Abcam (UK) | 40 μM | × |
| CBP30 | p300 | Cayman Chemical (USA) | 5 μM | × |
