# Supplementary material for: Histone acetyltransferase inhibition reverses opacity in rat galactose-induced cataract
Source: PLoS One. 2022 Nov 23;17(11):e0273868. doi: 10.1371/journal.pone.0273868 (PMC9683626; doi:10.1371/journal.pone.0273868)
Supplement: S3 Table — The column "ctrl-Galday4" indicates whether gene expression increased or decreased relative to that in the control after 4 days of incubation with galactose. The "Galday4-Day6" column shows whether gene expression increased or decreased from Day 4 to Day 6 of incubation with galactose. (DOCX) [file pone.0273868.s008.docx]

| **ctrl-Galday4** | **Galday4-Day6** | **No. of genes** | **Gene name(s)** |
| --- | --- | --- | --- |
| up | up | 1 | *Hspa1b* |
| up | down | 9 | *Tcp11l2 Rdh11 Rcbtb2 Nrip2 LOC100911253  LOC100362572 Hist1h2bh Gtpbp2 Bace1* |
| - | up | 9 | *Tprn Tgfb3 Sgpp1 Rtn4 Mir22  Ier3 Hamp Egr1 Cd44* |
| up | - | 993 | - |
