# Supplementary material for: Histone acetyltransferase inhibition reverses opacity in rat galactose-induced cataract
Source: PLoS One. 2022 Nov 23;17(11):e0273868. doi: 10.1371/journal.pone.0273868 (PMC9683626; doi:10.1371/journal.pone.0273868)
Supplement: S4 Table — This table shows the number of genes whose expression in each galactose sample increased or decreased relative to that in the control sample, and was decreased or increased by treatment with any one of three therapeutic agents. Significance was defined as P < 0.1, P < 0.05, and P < 0.01, and the number of each was noted. The same analysis was repeated for six galactose samples. The “Union” row shows the number of genes selected from any of the six galactose samples. The “Intersect” line shows the number of genes selected from all six galactose samples. (DOCX) [file pone.0273868.s009.docx]

| Galactose increases gene expression | | | |  | Galactose decreases gene expression | | | |
| --- | --- | --- | --- | --- | --- | --- | --- | --- |
|  | *P* < 0.1 | *P* < 0.05 | *P* < 0.01 |  |  | *P* < 0.1 | *P* < 0.05 | *P* < 0.01 |
| Galactose day4-1 | 139 | 87 | 34 |  | Galactose day4_1 | 148 | 86 | 44 |
| Galactose day4-2 | 149 | 90 | 32 |  | Galactose day4-2 | 117 | 71 | 17 |
| Galactose day4-3 | 153 | 98 | 48 |  | Galactose day4-3 | 95 | 51 | 16 |
| Galactose day6-1 | 143 | 82 | 26 |  | Galactose day6-1 | 142 | 82 | 26 |
| Galactose day6-2 | 171 | 112 | 44 |  | Galactose day6-2 | 116 | 52 | 17 |
| Galactose day6-3 | 160 | 91 | 44 |  | Galactose day6-3 | 102 | 48 | 19 |
| union | 421 | 248 | 109 |  | union | 412 | 221 | 77 |
| intersect | 25 | 14 | 3 |  | intersect | 14 | 9 | 2 |
